# Efficacy of modified team‐based learning in a flipped classroom for an acute‐care nursing course: A mixed‐methods study

**DOI:** 10.1002/nop2.1730

**Published:** 2023-03-23

**Authors:** Hiroaki Murata, Shoko Asakawa, Takao Kawamura, Hideki Yamauchi, Osamu Takahashi, Richard Henker

**Affiliations:** ^1^ National Defense Medical College Saitama Japan; ^2^ Faculty of Nursing and Medical Care Keio University Tokyo Japan; ^3^ School of Nursing at Narita International University of Health and Welfare Chiba Japan; ^4^ Toho University Tokyo Japan; ^5^ Graduate School of Public Health St. Luke's International University Tokyo Japan; ^6^ Internal Medicine St. Luke's International Hospital Tokyo Japan; ^7^ University of Pittsburgh Pittsburgh Pennsylvania USA

**Keywords:** critical thinking, nursing students, personal satisfaction, self‐learning, team‐based learning

## Abstract

**Aim:**

To test a modified team‐based learning approach on undergraduate learning outcomes in an acute‐care nursing course in Japan.

**Design:**

Mixed‐methods.

**Methods:**

Students worked on three simulated cases, engaged in pre‐class preparation, completed a quiz and engaged in group work. We collected data on team approach, critical‐thinking disposition and time spent in self‐learning at four time‐points: before the intervention and after each simulated case. Data were analysed using a linear mixed model, a Kruskal–Wallis test and a content analysis.

**Data sources:**

We recruited nursing students attending a mandatory course in acute‐care nursing at University A. Data were collected at four time‐points between April and July 2018. Data from 73 of 93 respondents were analysed.

**Results:**

Team approach, critical thinking and self‐learning all increased significantly across the time‐points. Four categories emerged from students' comments: ‘achievement of teamwork’, ‘sense of learning efficacy’, ‘satisfaction with course approach’ and ‘issues related to course approach’. The modified team‐based learning approach led to improvements in team approach and critical‐thinking disposition across the course.

**Conclusion:**

Incorporating team‐based learning into the curriculum not only contributes to team building but is also effective as a teaching method to improve student learning.

**Implications for the profession and/or patient care:**

The intervention led to improvements in team approach and critical‐thinking disposition across the course. The educational intervention also led to more time for self‐learning. Future studies should include participants from various universities and evaluate the outcomes over a longer period.

## 1 INTRODUCTION

In the model core curriculum for nursing education in Japan, published by the Ministry of Education, Culture, Sports, Science and Technology (MEXT) in 2017, the abilities required of nurses are ‘nursing knowledge and practical nursing skills’ and ‘[ability to] work in inter‐professional teams of healthcare, medical care, and welfare professionals’. These requirements have created a growing need to ensure the development of nursing curricula that equip nurses with the critical‐thinking skills necessary for evidence‐based nursing practice and to encourage a team approach for inter‐professional collaboration.

MEXT also noted that nursing students spend too little time studying outside of regular classes (Central Council for Education, 2012), and they stressed that nursing education should cultivate motivation for lifelong learning. To this end, the goal of ensuring that nursing students practice self‐directed learning and apply their knowledge and skills autonomously was set (MEXT, 2017). Thus, in their efforts to help students master practical nursing skills, nurse educators are to use paedagogical strategies that facilitate self‐driven learning.

Considering other recommendations regarding pedagogy, MEXT now also urges educators to shift away from teacher‐centred, lecture‐based teaching and towards learner‐focused active‐learning approaches. MEXT adopted this stance in 2012, following a report from its permanent advisory council, the Central Council for Education (2012), titled ‘Toward a qualitative shift in higher education that will build a new future: For universities that encourage lifelong learning and independent thinking’.

One form of active learning is team‐based learning (TBL), developed by Larry K. Michaelsen in the 1970s. TBL attempts to extend the effects of small‐group learning to large‐sized classes. This active‐learning approach involves pre‐class preparation, in‐class learning, and post‐class development (Michaelsen et al., 2008). The United States version of TBL has a three‐step cycle: preparation, in‐class readiness assurance testing and application‐focused exercises, consisting of team‐based application exercises (learners work in teams to solve application problems) and peer evaluation (learners evaluate each other's work; Davidson et al., 2014; Michaelsen et al., 2008; Parmelee et al., 2012).

The Anglosphere literature suggests that: Learners generally have a favourable impression of TBL; TBL is associated with learner satisfaction (Considine et al., 2021; Dearnley et al., 2018); and TBL is effective for improving self‐directed attainment (Alberti et al., 2021; Dearnley et al., 2018), practice development (Dearnley et al., 2018), problem solving (Kim et al., 2016), communication skills (Alberti et al., 2021; Lang et al., 2019) and teamwork (Considine et al., 2021). However, it remains unclear whether TBL can serve as a paedagogical approach for facilitating what MEXT requires: self‐directed learning for mastery of practical nursing skills, independence and autonomy. Furthermore, few studies have objectively measured the learners' team approach (Considine et al., 2021). In addition, there have been reports of disadvantages such as learner dissatisfaction and the burden of peer evaluation, which is commonly performed in TBL, on the course director. It is necessary to consider a method that is more acceptable to students (Farland et al., 2013).

## 2 BACKGROUND

In Japan, TBL and flipped classrooms (FCs) were introduced into nursing education in the 2000s, triggered by the recommendation of active learning by MEXT. Reports suggest that introducing TBL leads to improvements in learners' knowledge (Funabashi et al., 2017) and self‐efficacy (Nagasawa et al., 2019).

The FC approach, a type of blended learning, is an instructional strategy that replaces in‐class lecture with individual work completed on their own at home. Instead of being given a lecture from teachers, students work on problem solving through individual and group work while interacting with teachers or classmate during class (Missildine et al., 2013). This pedagogy is effective for improving nursing students' knowledge, skills, attitudes, self‐driven learning and critical‐thinking skills (Tan et al., 2017). Currently, 83% of the Japanese population and 98% of people in their teens and 20s use the Internet via smartphones and personal computers. (Ministry of Internal Affairs and Communications [MIC], 2020). Thus, the information and communication technologies (ICT) environment for digital learning, particularly mobile learning, is getting established for education in Japan. However, in‐class lecture‐based learning, a style in which students learn from one‐way lectures, is still one of Japan's most broadly used teaching methods in nursing education.

We focused on ‘peer evaluation’ as one of the components of TBL as a factor that hinders effective TBL implementation among Japanese students. Some reports have highlighted concerns regarding the use of TBL for Japanese students. One study reported that learners were reluctant to engage in peer evaluation (Saito & Saito, 2013), while in another study, learners said that their individual efforts were not fairly appraised (Nagasawa et al., 2018). Because Japanese people have traditionally valued harmony and homogeneity, and avoided unnecessary friction between people (Konishi et al., 2009), some Japanese learners could be uncomfortable with peer evaluations. Other researchers reported that students disliked peer evaluation owing to their concern about relationships with group members (Levine, 2008) and the possibility of experiencing a sense of socio‐emotional discomfort (Topping et al., 2000). These concerns imply that for TBL to be adopted as part of the core nursing curriculum in Japan, it is necessary to modify it to make it better suited to the needs of Japanese learners and then verify the effects of this modified version.

This study examined the effects of an intervention consisting of M‐TBL delivered in a FC on students' team approach, critical‐thinking disposition and time spent in self‐learning. Participants were undergraduate nursing students in an acute‐care nursing course.

## 3 THIS STUDY

### 3.1 Research design

When participants are students, it is ethically and practically undesirable to conduct randomized controlled trials in TBL; rather, one should design using qualitative and mixed methodologies without making a non‐control group (Considine et al., 2021). Therefore, a one‐group pretest–posttest design was used. Additionally, this study adopted a concurrent mixed‐methods design using a self‐reported survey. In this design, quantitative data related to team approach, critical‐thinking disposition and self‐learning are collected and analysed concurrently with the qualitative data to triangulate the findings, allowing mutual corroboration or reinforcement that enriches understanding.

### 3.2 Nomenclature

#### 3.2.1 Team approach

We adopted the definition of ‘team approach’ in Iioka et al., (2016): supportive teamwork in which each team member actively engages in the decision‐making process and performs their respective role collaboratively so the team can accomplish complex tasks.

#### 3.2.2 Critical thinking

We adopted the definition of ‘critical thinking’ used by Tokiwa et al., (2010): A pattern of reflective thinking that has elements of knowledge, skills and attitudes. A person engaging in critical thinking consciously examines appropriate criteria and evidence in an inferential process and focuses on deciding what to believe and how to act on this belief.

#### 3.2.3 Critical‐thinking disposition

We adopted the definition of ‘critical‐thinking disposition’ used by Tokiwa et al., (2010): an element of critical thinking, willingness to practice critical thinking, a disposition that facilitates or supports the knowledge and skills used in critical thinking.

#### 3.2.4 M‐TBL

M‐TBL is TBL without the peer evaluation step, which is the final step in the original (US) version of TBL. Peer evaluation was removed from M‐TBL, based on Saito and Saito (2013) report that Japanese nursing students were reluctant to engage in this step.

### 3.3 Method

#### 3.3.1 Participants and sample size

We recruited 105 third‐year undergraduate nursing students attending a mandatory course in acute‐care nursing at University A. Data were collected at four time‐points between April and July 2018, during one course term. The primary outcome was the effects of perception on team approach peer evaluation. No statistical sample size calculations were conducted because the sample size was determined by the class. However, a sample size of 26 students per group was given post hoc power (1–β) of 80%, using an effect size of 0.40 and a significance level of *p* < 0.05. The estimated sample size was 105. Considering a response rate of 50% based on previous research and daily reaction in the class, we calculated the total sample size as 52 (26 students per group). A power calculation indicated that the sample size was sufficient to assess our primary outcome, the effects of perception on team approach peer evaluation.

#### 3.3.2 Description of the intervention

Acute‐care nursing, the course to which the M‐TBL intervention was applied, is traditionally a lecture‐based class and is a mandatory course consisting of 15 classroom meetings. The syllabus states that the goal of the course is for students to master the team approach and a critical‐thinking disposition, in addition to knowledge. The course focused on cultivating core competencies in the nursing process. Based on this approach, students engaged in three simulated cases set by the teacher. The three cases were paper‐based (as opposed to virtually simulated). The first case involved gastrointestinal disease, the second cardiovascular disease and the third neurological disease. When setting the order of the cases, the teacher coordinated with other teachers so the cases would occur concurrently with the relevant learning content, which included ‘disease and therapeutics’ and ‘pharmacology’. The students were divided into 21 groups of five, based on existing reports suggesting that groups of five to seven are optimal (Michaelsen et al., 2008; Parmelee et al., 2012). Students were randomly allocated to each group. The intervention procedure is described below.

##### Digitally powered FC format (pre‐class preparation)

With the introduction of M‐TBL, we applied the FC format because of its structural affinity (Jakobsen & Knetemann, 2017) for encouraging self‐driven learning and learner autonomy (Tan et al., 2017). For the pre‐class preparation, which started one or 2 weeks before the group discussion, students viewed the original lecture video made by the teacher. In the video, the teacher used a PowerPoint presentation to communicate the basic knowledge that students would need to engage in group work. The video was streamed using video‐on‐demand technology. Each video lasted approximately 30–45 min. The students accessed and viewed the video in their own time.

##### Individual readiness assurance test (IRAT)

The IRAT is a short test that was taken by students before class meetings. The tests consisted of approximately five questions related to the on‐demand lectures in the videos. The questions were designed to confirm students' readiness for class meetings, and as such, were based on ‘knowledge’ and ‘comprehension’, the first two categories of Bloom's taxonomy (Engelhart et al., 1956), which is applied in medical education. Performance in the IRAT accounted for 30% of students' final grades for the course.

##### Group work and evaluation of group work

After the test, students gathered into their groups, worked on the case material, assessed the case, developed a nursing plan and then delivered a group presentation during class meetings. With prompting from the teacher, the group members worked together to resolve the questions and doubts that had arisen during the group discussion. For the final evaluation of the group work, three teachers used unified rubrics to score the team's case materials, case assessment and nursing plan. This score accounted for 20% of students' final grade.

#### 3.3.3 Data collection method

**FIGURE 1:**
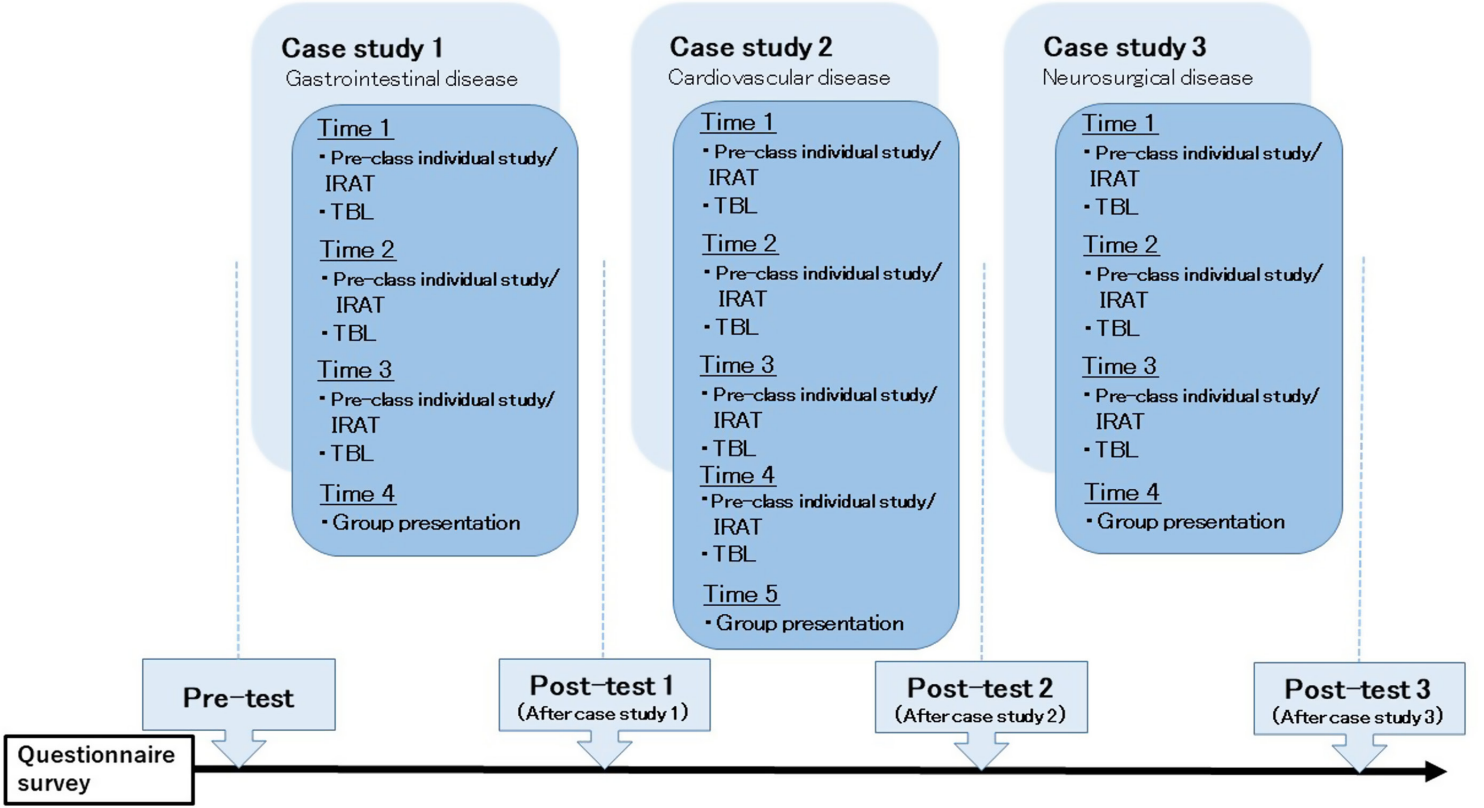
Course development and timing of data collection.

Students completed a self‐reported survey at four time‐points: at the start of the intervention (pre‐test) and at the end of each of the three cases (post‐test 1, 2 and 3; Figure 1). The self‐reported surveys were completed on SurveyMonkey, an online survey tool accredited as compliant with international data security standards.

#### 3.3.4 Survey items

The authors designed a questionnaire survey that consisted of 50 questions measuring the following items: team approach, critical‐thinking disposition, age, sex, time spent in self‐learning (including pre‐class preparation), opinion on peer evaluation (whether it should be included) and opinion on group methods for learning (whether the groups should be changed). The questionnaire included a space at the end to allow respondents to write comments freely.

The team approach was measured using the Team Approach Assessment Scale (TAAS) developed by Iioka et al., (2016). The TAAS, which applies to a healthcare team in which nurses engage, measures the extent that respondents believe that their team embodies the team approach. The scale has 26 items (Cronbach's alpha = 0.97) and four subscales: team function, team communication, membership and contribution to the team. Each item is rated on a 4‐point Likert type scale from ‘strongly disagree’ to ‘strongly agree’.

Critical‐thinking disposition was measured using Tokiwa et al. (2010) scale for critical‐thinking disposition in basic nursing education. This scale has 15 items (Cronbach's alpha = 0.9) and five subscales: scepticism, groupthink‐like behaviour, tenacity, inquisitiveness and confidence in one's logical thought. Each item was rated on a 5‐point scale from ‘very often” to “never’.

For time spent in self‐learning, to reduce the difficulty of inputting the data, participants were presented with an ordinal scale consisting of only four answer options: ‘less than 30 minutes’, ‘30 minutes to an hour’, ‘one to two hours’ and ‘more than two hours’.

#### 3.3.5 Analytical method

##### Quantitative data

Data were collected from the 21 groups at four time‐points. A linear mixed model was used to analyse the team approach and critical‐thinking disposition.

For team approach, ‘team approach’ was input as the dependent variable, ‘group’ and ‘time‐point’ were input as fixed effects, and ‘participant’ was input as a random effect. Team approach was also analysed in relation to opinions on peer evaluation. Specifically, while ‘participant’ was retained as a random effect and ‘team approach’ as the dependent variable, the group's opinion on peer evaluation (for or against) was input as a fixed effect. A group was recorded as in favour of peer evaluation if at least one member of the group reported, at the time‐point in question, that they believed peer evaluation was necessary. Otherwise, if every single member was against it, the group was documented as against peer evaluation.

For critical‐thinking disposition, ‘critical‐thinking disposition’ was input as the dependent variable, ‘time‐point’ was input as a fixed effect and ‘participant’ was input as a random effect.

The Bonferroni test was used to conduct multiple comparisons and tests for the main effects in the linear mixed model.

For time spent in self‐learning, the Kruskal–Wallis test was used to compare the data across time‐points, and multiple comparisons were conducted using the Bonferroni test.

##### Qualitative data

The qualitative data were the free comments of the respondents, in which they described their experience with M‐TBL. Data were analysed using content analysis to identify themes in the data. The procedure is described as follows:

1. The comments were read repeatedly to identify the textual contexts for statements about M‐TBL.
2. The extracted contexts were encoded to preserve their semantic content.
3. The codes were compared based on similarities and differences in the descriptive content. Similar codes were grouped together to form the subcategories. Each subcategory was named.
4. The subcategories in (3) were compared concerning their names, constituent codes and the similarities and differences in their descriptive content. Similar subcategories were grouped together to form categories, after which each category was named.

To ensure the validity of this analysis, the following procedure was performed: First, each author, working separately, encoded the contexts. Next, the authors came together and compared their findings, re‐examined their coding and discussed inter‐author discrepancies in the codes until consensus was reached. This process was repeated for subcategories and categories; the authors, working separately, formed the subcategories and categories, and then they came together to compare findings and resolve discrepancies.

#### 3.3.6 Ethical considerations

To prevent coercive participation, students were informed verbally and in writing that their decision to participate or not participate would not affect their academic results. Participants were deemed to have consented if they responded to the survey after receiving this information. To protect their privacy, participants could contact a third party (a party not connected with the implementation or evaluation of the course) to withdraw their participation anonymously. Additionally, the survey data were collected online with an anonymized identification number assigned to each participant to ensure that no participant could be personally identified from the data they provided. The study was approved by the ethics committee of the authors' affiliate university (no. 17‐Io‐183).

## 4 RESULTS

### 4.1 Sample characteristics

The surveys were distributed to 105 students. Responses were obtained from 93 students representing 21 groups. Of these, 20 respondents were removed from the sample because of missing values for major variables at all four time‐points. Thus, the final sample consisted of 73 students (70 women), representing 19 groups. The mean age of students was 20.3 (± 2.1) years.

### 4.2 Effects of intervention (M‐TBL)

#### 4.2.1 Team approach

Table 1 shows the results of the analysis using a linear mixed model. Regarding the scores for team approach among the 19 groups, no interaction was observed between ‘group’ and ‘time‐point’ (*F*[54, 90.427] = 1.240, *p* = 0.181). The scores did not vary significantly between the groups (*F*[18,49.596] = 0.738, *p* = 0.756). However, the scores increased significantly across time‐points (*F*[3,94.199] = 8.848, *p* < 0.001). This increase was particularly conspicuous at post‐test 2 (after the second case study) and post‐test 3 (after the third case study, marking the end of the course).

**TABLE 1:** Scores across the four time‐points.

Item measured	Fixed effect (variables other than time‐point)	Time‐point				*p*‐value	Interaction
		Pre‐test (SE)	Post‐test 1 (SE)	Post‐test 2 (SE)	Post‐test 3 (course end) (SE)		
Team approach	Group (1–19)	47.625 (1.697)	50.533 (1.641)	52.982 (1.454)*	55.340 (1.398)*	<0.001^a^	N/A
	Group's opinion on peer evaluation (for/against)	48.489 (1.521)	51.284 (1.548)	53.663 (1.283)*	55.984 (1.276)*	<0.001^b^	N/A
Critical‐thinking disposition	–	53.505 (0.955)	54.195 (1.007)	55.0813 (0.892)	55.960 (0.895)*	0.028	–

Note: *p < 0.05; change from pre‐test according to multiple comparison performed with Bonferroni test.

Abbreviations: N/A, not applicable; SE, standard error.

^a^ Based on mixed linear model with ‘group’ (1–19 [total of 73 participants]) and ‘time point’ as fixed effects and ‘participant’ as random effects.

^b^ Based on mixed linear model with ‘group's opinion on peer evaluation’ (for = 11 groups [45 participants], against = 8 groups [28 participants]) and ‘time point’ as fixed effects and ‘participant’ as random effects.

Regarding opinions on peer evaluation (Table 1), 11 groups (45 participants) were in favour of peer evaluation, while eight groups (28 participants) were against it. No interaction was found between the ‘group's opinion on peer evaluation’ and ‘time‐point’ in relation to the team approach scores (*F*[3142.749] = 0.775, *p* = 0.510). The group's opinion on peer evaluation had no significant impact on team approach scores (*F*[1,65.462] = 1.650, *p* = 0.203). In both sets of groups (for and against), team approach scores increased across time‐points (*F*[3142.749] = 8.891, *p* < 0.001). As with the 19‐group analysis, this increase was particularly noticeable at post‐tests 2 and 3.

#### 4.2.2 Critical‐thinking disposition

According to the linear mixed model (Table 1), critical‐thinking disposition increased significantly across time‐points (*F*[3162.467] = 3.112, *p* = 0.028). The increase was particularly noticeable at post‐test 3.

### 4.3 Time spent in self‐learning

**FIGURE 2:**
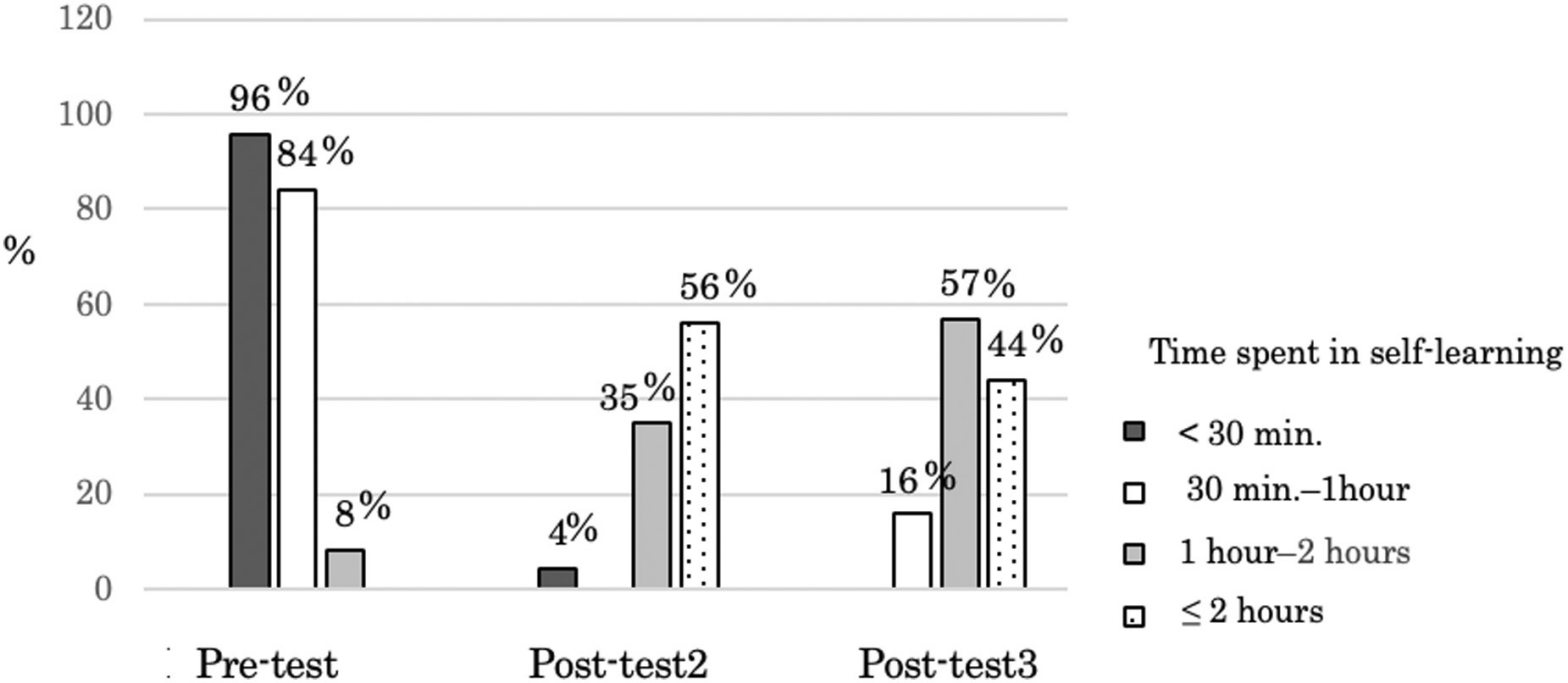
Time spent in self‐learning.

Figure 2 shows the results of the time spent on self‐learning. No results are shown for post‐test 1; the data for this time‐point were irretrievable owing to technical issues. The Kruskal–Wallis test revealed that the time spent in self‐learning was statistically significant (*p* < 0.001) at each time‐point. The Bonferroni test revealed that it was significantly higher (*p* < 0.001) at post‐test 2 and 3 than at pre‐test.

### 4.4 Student feedback

A content analysis was conducted on the free comments obtained at post‐test 1 and post‐test 3 but not for post‐test 2. There were not enough comments for post‐test 2 to identify categories at that time‐point.

Table 2 shows the categories and subcategories induced in the analysis of the comments at the two time‐points. Categories are indicated by double angle brackets (<<Category>>), while subcategories are indicated by single angle brackets (<Subcategory>). For each subcategory, the table shows examples of the statements. The categories and subcategories are described below.

**TABLE 2:** Attitudes towards the M‐TBL induced from free comments.

Time‐point	Category	Subcategory	Example of statement
Post‐case 1	Category 1: <<Sense of learning efficacy>>	<Sense of self‐directed learning efficacy>	I deepened my knowledge more than I would have done if the course had just used a lecture format. The group work was hard going, but it meant that I engaged with the learning more autonomously than I usually do.
		<Sense of efficacy that offsets heavy workload>	Frankly, the workload was heavy; but, by the same token, it gave me a sense of mastery.
		<Motivation raised by learning style>	The teacher in acute nursing made the learning easy for me to understand and provided well‐thought‐out material for pre‐class preparation. This delighted me and boosted my learning motivation.
	Category 2: <<Achievement of teamwork>>	<Gaining perspectives from other members>	Through the teamwork, I was introduced to perspectives that I had not considered.
		<Sense of team collaboration>	I think everyone is working hard to a certain level through teamwork.
	Category 3: <<Issues related to course approach>>	<Suggestions for course approach>	There should be a bit more time for group work in the class.
		<Troubled by disharmony in team>	Most members got on, but there were some members who did not cooperate no matter what approach we took. I do not know how to deal with this.
	Category 4: <<Satisfaction with course approach>>	<Satisfaction with course approach>	This format was a first for me. I appreciated being able to do the pre‐class work (viewing the video) at my own pace.
Post‐case 3	Category 1: <<Sense of learning efficacy>>	<Acquisition of thinking skills and knowledge>	The learning style was just right for me. It helped me gain thinking skills and knowledge.
		<Sense of efficacy that offsets heavy workload>	The work was tough, but I think it will prove useful.
		<Overcoming of poor confidence>	I was unsure at first, but I now think the learning has been very good. I previously felt unconfident in the nursing process, but now I love it.
	Category 2: <<Achievement of teamwork>>	<Achievement of teamwork>	I was very happy with the group work. Sharing ideas in the group helped us assess the case and develop a better plan.
	Category 3: <<Issues related to course approach>>	<Sense of unfairness at contribution>	Group work is very valuable when the members engage positively, but it is hard going if some members do not pull their weight or if one member has to shoulder all the work. Hence, each member might need to be evaluated by the other members. It is natural that some groups will do better than others, but it strikes me as really unfair that students who worked really hard should get a bad score because other members in their group did not contribute much.
		<Suggestions for course approach>	It was good to do the pre‐class prep before the group work, but I think I could have understood the content better if there had been a lecture from the teacher following the pre‐class prep.
	Category 4: <<Satisfaction with course approach>>	<Satisfaction with course approach>	I really appreciated the class itself, the intuitive explanation and the way the class developed.

#### 4.4.1 Post‐test 1

From the data at post‐test 1, eight subcategories were induced. From these, four categories were identified. The first category was <<sense of learning efficacy>>. This category incorporated three subcategories: <sense of self‐directed learning efficacy>, <sense of efficacy that offsets heavy workload> and < motivation raised by learning style>. <Sense of self‐directed learning efficacy> described the belief that the intervention facilitated learners' autonomy and produced good learning outcomes. <Sense of efficacy that offsets heavy workload> described students' belief that heavy workload was worth the sense of efficacy it promoted.

The second category was <<achievement of teamwork>>, which included two subcategories: <sense of team collaboration> and < gaining perspectives from other members>. The former refers to how members recognized each other's efforts to engender a sense of collaboration within the team, while the latter refers to the sharing of perspectives regarding teamwork between different team members.

Not all students managed to get on to other team members. One statement referring to team problems was grouped under the subcategory <troubled by disharmony in team>, which itself was grouped under the category << issues related to course approach>>.

#### 4.4.2 Post‐test 3

Seven subcategories were induced from the data at post‐test 3 and combined to form four categories like those induced at post‐test 1. The first category was <<sense of learning efficacy>>, which included subcategories <acquisition of thinking skills and knowledge> extracted from M‐TBL. It described the view that the intervention helped the person acquire the knowledge and thinking skills necessary to engage in the case. The second subcategory was <overcoming of poor confidence>, which described the view that the intervention helped the person to overcome their lack of confidence in understanding the nursing process, and facilitating knowledge and skills.

The second category was <<achievement of teamwork>>. This category described the belief that smooth teamwork, in addition to giving a sense of accomplishment, proved effective in helping the person assess the case and develop a nursing plan. Similar to post‐test 1, the data yielded the category <<issues related to course approach>>. However, this category included a new subcategory, <sense of unfairness at contribution>, which described a sense that some members contributed more than others towards the team's final product, that the workload was distributed unevenly and that the evaluation did not consider members' individual contributions.

## 5 DISCUSSION

The quantitative analysis indicated that team approach, critical‐thinking disposition and time spent in self‐learning increased significantly across time‐points. The qualitative analysis yielded four categories: <<achievement of teamwork>>, <<sense of learning efficacy>>, <<satisfaction with course approach>> and < <issues related to course approach>>. Below, we discuss the quantitative and qualitative findings regarding team approach, critical‐thinking disposition and time spent in self‐learning.

### 5.1 Team approach

The quantitative analysis indicated that team approach increased significantly across the course, irrespective of opinion on peer evaluation. Moreover, previous studies have shown higher post‐test scores than pre‐test scores in teamwork (Goolsarran et al., 2018; Roh et al., 2020). The qualitative analysis did reveal <<issues related to course approach>>: at post‐test 1, some participants were < troubled by disharmony in team>, meaning that at post‐test 3, some had a < sense of unfairness at contribution>. However, participants continued to perceive the effects of <<achievement of teamwork>> throughout the intervention, including <gaining perspectives from other members> and < a sense of team cohesion>. Thus, taken together, the quantitative and qualitative findings suggest that although some participants said concerned about team member relationships and apparent unfairness, their team approach strengthened as the intervention progressed. These results indicate that M‐TBL is a useful educational method for nursing students as part of a method to enhance the team approach, which is an essential skill for nurses working in collaboration with multiple professions.

However, it is necessary to reduce the sense of unfairness in members' contributions which may occur in TBL without using peer evaluation system. From the qualitative result, we believe that there are two types of students who are creating differences in contribution that may create a source of dissonance in teamwork: ‘students who cut corners’ and ‘students who are not good at being proactive’.

The solution may require a more robust evaluation system, one that discourages ‘students who cut corners’, which is as known as ‘social loafing’ (Gabelica et al., 2022). One possible strategy for preventing members from losing their motivation to contribute and exert effort could be to assess the contributions of each member and the group's overall work. As this strategy may increase faculty workload, it might be a good idea to integrate digital technology into an evaluation system that actively utilizes ICT.

Another issue to consider is psychological safety. Digital natives may be less accustomed to offline, face‐to‐face interpersonal scenarios than other demographics, which may create passivity in teamwork. If students struggle to master the teamwork skills necessary for inter‐professional collaboration, the faculty should provide a setting for group work that creates psychological safety, as a sense of trust encourages better communication, which enhances team efficacy, both concerning learning and the production of work (Park & Kim, 2021). For example, before the class begins, the teacher can announce rules and require students to pledge their compliance with them. The rules could include allowing team members to express their ideas freely or refraining from divulging members' ideas to people outside the group. It is essential to recognize each other as team members to collaborate with rather than team members to be monitored and evaluated.

### 5.2 Critical‐thinking disposition

Our finding that the M‐TBL intervention improved critical‐thinking disposition is consistent with the finding of Tan et al. (2017), who illustrated that a FC format improves critical‐thinking skills. The qualitative analysis provides further evidence for the above. The evidence is the in‐vivo code ‘(the learning style) helped me gain thinking skills and knowledge’, which formed part of the category <<sense of learning efficacy>>.

Critical‐thinking disposition forms an essential part of the critical‐thinking process, in that it facilitates the knowledge and skills involved in critical thinking. This disposition has become more important in the new normal of nursing, a nursing milieu that features higher levels of diversity and uncertainty because of rapid advances in nursing technology and the rise of new diseases such as COVID‐19. Against this backdrop, it is essential for nurses to take a problem‐solving approach, in which they critically evaluate the criteria and evidence for prospective actions and collaborate with and consider the ideas of others. The critical‐thinking process resembles the nursing process and, as such, is essential for ensuring effective nursing practice (Kusumi, 2015; Suzuki et al., 2015). One study found that people with higher scores for critical thinking had a stronger propensity for using scientific evidence (Futami et al., 2019). In an age when people can easily find information online, it is essential that nurses enhance their critical‐thinking disposition and skills while in nursing school.

### 5.3 Time spent in self‐learning

The quantitative analysis indicated that the time students spent on self‐learning increased during the course. Moreover, the qualitative analysis suggested that the intervention was effective in supporting independent study. Specifically, as part of their <<sense of learning efficacy>>, students said a < sense of efficacy that offsets heavy workload>, a < sense of self‐directed learning efficacy> and a sense of <overcoming of poor confidence>. It is considered that this M‐TBL incorporating the FC has a certain effect. As a further testament to the impact of the intervention, students retained their <<sense of learning efficacy>> throughout the course (the impact was present post‐test 3 and post‐test 1).

This finding is consistent with the review by Tan et al. (2017), which suggested that a FC may improve self‐driven learning. One possible reason the intervention supported self‐learning was that it required the use of digital media that students could view whenever and wherever they wanted. The intervention also used IRATs related to the on‐demand lecture in the videos, ensuring that students could only participate in the class if they viewed the video. Another motivating factor was that poor performance in an IRAT negatively affected students' individual evaluations. Since the advancement of digitalization has been led, incorporating personalized learning through digital materials into the teaching format will allow students to continue learning even when minimal face‐to‐face instruction is required, as in the recent COVID‐19 pandemic.

Although the university establishment standards, instituted by MEXT through ministerial order, stipulate that mandatory classes should involve a sufficient element of self‐study outside class hours (MEXT, 2019), reports suggest that students spend ‘no time at all’ or ‘less than 30 minutes before or after the class’ in such self‐study (Kanayama et al., 2016; Yamauchi, 2019). When evaluated in the context of this situation, our intervention proved successful in that it encouraged students to spend plenty of time in self‐learning by providing content that could be viewed on demand and by requiring students to view the material as a prerequisite for participating in the class.

Another factor to consider is the time and effort required of teachers in developing the educational materials, which is likely to have boosted students' motivation, as is evident from the qualitative data—specifically, the in‐vivo code reading, ‘the teacher…prepared well‐thought‐out material for pre‐class preparation (and this) boosted my learning motivation’, which was part of the subcategory <motivation raised by learning style>.

### 5.4 Limitations

This study has certain limitations. The loss of data for the variable self‐study time for post‐test 1 owing to technical issues may have compromised our understanding of the impact of TBL on self‐study time. Moreover, all participants were from the same university in Japan. Future studies should include participants from various universities. Lastly, the evaluation was performed only for a limited period. In future, it will be necessary to evaluate the outcomes over a longer period.

## 6 CONCLUSION

The MEXT in Japan published an additional requirement for healthcare providers to have the ability to work as part of an inter‐professional team. Incorporating TBL into the curriculum not only contributes to team building but is also effective as a teaching method to improve student learning. We tested an educational intervention consisting of M‐TBL (TBL without the peer evaluation component) among third‐year undergraduate nursing students from a university in Japan. The intervention led to improvements in team approach and critical‐thinking disposition across the course. The educational intervention also led to more time in self‐learning.

## AUTHOR CONTRIBUTIONS

HM oversaw the study plan, data collection and overall article writing. SA, TK, HY, OT (expert statistician) and RH contributed to the conception and design of the work. SA, TK and RH also contributed to interpreting data and revising this article critically. All authors approved the version to be submitted. We have read and understood your journal's policies on copyright, ethics, etc., and believe that neither the article nor the study violates any of these. All those entitled to authorship are listed as authors. All authors meet the authorship criteria.

## FUNDING INFORMATION

This work was supported by Japan Society for the Promotion of Science, Tokyo, Japan (KAKENHI Grant‐in‐Aid for Early‐Career Scientists, Research Project No: 20 K19044).

## CONFLICT OF INTEREST STATEMENT

No conflicts of interest are declared by the authors.

## ETHICS STATEMENT

The study was approved by the ethics committee of the international university of Health and Welfare (No. 17‐Io‐183).

## Data Availability

The data that support the findings of this study are available on request from the corresponding author. The data are not publicly available due to privacy and ethical restrictions.
